# Association between SARS-CoV-2 infection and self-harm: Danish nationwide register-based cohort study

**DOI:** 10.1192/bjp.2022.194

**Published:** 2023-04

**Authors:** Annette Erlangsen, Ping Qin, Trine Madsen, Keith Hawton, Merete Osler, Carsten Hjorthøj, Michael E. Benros, Steen Ethelberg, Kåre Mølbak, Thomas Munk Laursen, Merete Nordentoft, Sandra Feodor Nilsson

**Affiliations:** Danish Research Institute for Suicide Prevention, Mental Health Centre Copenhagen, Copenhagen, Denmark; Copenhagen Research Center for Mental Health, Mental Health Centre Copenhagen, Copenhagen, Denmark; Department of Mental Health, Johns Hopkins Bloomberg School of Public Health, Baltimore, Maryland, USA; and Center of Mental Health Research, Australian National University, Canberra, Australia; National Center for Suicide Research and Prevention, Institute of Clinical Medicine, University of Oslo, Oslo, Norway; Danish Research Institute for Suicide Prevention, Mental Health Centre Copenhagen, Copenhagen, Denmark; Copenhagen Research Center for Mental Health, Mental Health Centre Copenhagen, Copenhagen, Denmark; and Section of Epidemiology, Department of Public Health, Faculty of Health and Medical Sciences, University of Copenhagen, Copenhagen, Denmark; Centre for Suicide Research, University of Oxford, Oxford, UK; and Oxford Health NHS Foundation Trust, Oxford, UK; Center for Clinical Research and Prevention, Bispebjerg & Frederiksberg Hospitals, Copenhagen, Denmark; and Section of Epidemiology, Department of Public Health, Faculty of Health and Medical Sciences, University of Copenhagen, Copenhagen, Denmark; Copenhagen Research Center for Mental Health, Mental Health Centre Copenhagen, Copenhagen University Hospital, Hellerup, Denmark; and Section of Epidemiology, Department of Public Health, Faculty of Health and Medical Sciences, University of Copenhagen, Copenhagen, Denmark; Copenhagen Research Center for Mental Health, Mental Health Centre Copenhagen, Copenhagen, Denmark; and Department of Immunology and Microbiology, University of Copenhagen, Copenhagen, Denmark; Infectious Disease Epidemiology & Prevention, Statens Serum Institut, Copenhagen, Denmark; and Department of Global Health, Faculty of Health and Medical Sciences, University of Copenhagen, Copenhagen, Denmark; Epidemiological Infectious Disease Preparedness, Statens Serum Institut, Copenhagen, Denmark; and Department of Veterinary and Animal Science, University of Copenhagen, Copenhagen, Denmark; National Centre for Register-Based Research, Aarhus University, Aarhus, Denmark; Danish Research Institute for Suicide Prevention, Mental Health Centre Copenhagen, Copenhagen, Denmark; Copenhagen Research Center for Mental Health, Mental Health Centre Copenhagen, Copenhagen, Denmark; and Institute of Clinical Medicine, University of Copenhagen, Copenhagen, Denmark; Copenhagen Research Center for Mental Health, Mental Health Centre Copenhagen, Copenhagen University Hospital, Hellerup, Denmark

**Keywords:** Suicide attempt, epidemiology, SARS-CoV-2, self-harm, COVID-19

## Abstract

**Background:**

Case studies have linked SARS-CoV-2 infection to suicidal behaviour. However, conclusive evidence is lacking.

**Aims:**

To examine whether a history of SARS-CoV-2 infection or SARS-CoV-2-related hospital admission was associated with self-harm in the general population and in high-risk groups.

**Method:**

A cohort design was applied to nationwide data on all people aged ≥15 years and living in Denmark between 27 February 2020 and 15 October 2021. Exposure was identified as having had a positive SARS-CoV-2 PCR test, and further assessed as SARS-CoV-2-related hospital admission. Rates of probable self-harm were examined using adjusted incidence rate ratios (aIRRs). The following subgroups were identified: (a) lower educational level, (b) chronic medical conditions, (c) disability pension, (d) mental disorders, (e) substance use disorders, and history of (f) homelessness and (g) imprisonment.

**Results:**

Among 4 412 248 included individuals, 260 663 (5.9%) had tested positive for SARS-CoV-2. Out of 5453 individuals presenting with self-harm, 131 (2.4%) had been infected. Individuals with a history of a positive SARS-CoV-2 test result had an aIRR for self-harm of 0.86 (95% CI 0.72–1.03) compared with those without. High rates were found after a SARS-CoV-2-related hospital admission (aIRR = 7.68; 95% CI 5.61–10.51) or a non-SARS-CoV-2-related admission (aIRR = 10.27; 95% CI 9.65–10.93) versus non-infected and not admitted. In sensitivity analyses with a more restrictive definition of self-harm, a positive PCR test was associated with lower rates of self-harm.

**Conclusions:**

Individuals with a PCR-confirmed SARS-CoV-2 infection did not have higher rates of self-harm than those without. Hospital admission in general, rather than being SARS-CoV-2 positive. seemed to be linked to elevated rates of self-harm.

According to case reports, being infected with SARS-CoV-2 might be associated with suicidal ideation or self-harm (intentional non-fatal self-poisoning or self-injury).^1–3^ Although large-scale studies of SARS-CoV-2 infection and self-harm are lacking, general infections have previously been linked to increased suicide risk.^4^ Suicidal ideation could also arise during periods of SARS-CoV-2 infection-related distress, for instance after isolation, quarantine or experiences of trauma, which have been reported frequently by SARS-CoV-2-positive individuals.^1,5^ Moreover, infections, and the activation of the immune system and systemic inflammation, related to SARS-CoV-2 have been associated with increased risk of mental disorders.^6,7^ Also, individuals with hospital contacts for SARS-CoV-2 have been found to have higher risks of mental disorders, such as mood and anxiety disorders, in comparison with individuals admitted to hospital for other reasons, for instance skin infection and fracture of large bones.^8^

## High-risk groups

Individuals with chronic medical conditions and mental disorders and also homeless or incarcerated people are all known to have higher rates of suicide.^9–12^ Lower SARS-CoV-2 vaccination rates have been found in these groups, implying an increased probability of a complicated course of any SARS-CoV-2 infection.^13^ Indeed, higher occurrences of SARS-CoV-2-related morbidity and mortality have been reported for all of these groups compared with the general population.^14^ During the COVID-19 pandemic, higher levels of psychological distress have been reported for several of these groups as well as for individuals of lower educational level.^15^ In addition to people with chronic medical conditions or mental disorders and those belonging to socially marginalised groups, older adults may be disproportionately affected by psychological distress when infected with SARS-CoV-2.^16^ It remains to be examined whether these high-risk groups may experience higher rates of self-harm after a SARS-CoV-2 infection, for instance owing to limited and interrupted access to ongoing and new treatments as well as to general support for other problems, such as social welfare.

The aim of the current study was to examine whether individuals after having tested positive for SARS-CoV-2 had higher rates of self-harm than individuals with no such history. Furthermore, we aimed to analyse whether people with (a) lower educational level, (b) chronic medical conditions, (c) disability pension, (d) mental disorders, (e) substance use disorder, or a history of either (f) homelessness or (g) imprisonment and who had tested positive for SARS-CoV-2 had higher rates of self-harm compared with peers with no history of a SARS-CoV-2 infection. Until the end of 2021, Denmark had one of the highest rates of PCR testing for SARS-CoV-2 in the world.^17^ It also has national administrative registries, which enable excellent individual-level linkage of data records.^18^ Linking information on individual PCR tests with other national individual-level data records provides a unique opportunity for conducting studies with high validity.

## Method

### Study design and data sources

A cohort design was applied to longitudinal, nationwide, register-based data on all people who were living in Denmark between 27 February 2020 and 15 October 2021. The start date marked the first confirmed SARS-CoV-2 case in Denmark^19^ and the end of follow-up was defined by the recency of data. Information on all residents living in Denmark was obtained from the Danish Civil Registration System.^20^ Using the unique personal identification number assigned to all individuals, a linkage to data from the following registers was facilitated: the Danish Microbiology Database,^21^ the Population Education Register,^22^ the National Patient Register,^23^ the Psychiatric Central Research Register,^24^ the National Prescription Registry,^25^ the Homeless Register^11^ and the Central Criminal Register.^26^ The National Patient Register and the Psychiatric Central Research Register provided dates and diagnoses from for all medical in-patient contacts since 1994 and all psychiatric in-patient contacts since 1969 respectively. Out-patient and emergency department contacts have been recorded since 1995 in both registers. Sociodemographic information, including socioeconomic status, was obtained from Statistics Denmark.

### Study population

All individuals who lived in Denmark and were 15 years of age or older, i.e. were born on 15 October 2006 or earlier, were included. The following subgroups, which have been suggested as being vulnerable to SARS-CoV-2 infection, were identified: people who had (a) primary school or less education, (b) chronic medical conditions, (c) disability pension, (d) a history of mental disorder, including severe mental illness, (e) a history of a substance use disorder, (f) history of homelessness and (g) history of imprisonment. Information on highest obtained education was collected from the Population Education Register and based on the status in September 2019 (Supplementary Table 1, available at https://dx.doi.org/10.1192/bjp.2022.194). Persons who had been approved for disability pension prior to 2020 were identified through their socioeconomic status. People with chronic medical conditions were identified using diagnoses for 31 medical conditions recorded according to ICD-10 in the National Patient Register^27^ and which had been diagnosed prior to 27 February 2020. In addition, records of medication prescribed for chronic medical conditions were obtained from the National Prescription Registry (Supplementary Table 2).^24,28^ Severe mental disorder was defined as having been diagnosed with schizophrenia, bipolar disorder or depressive disorder at some point after 1969 and recorded in the Psychiatric Central Research Register. Persons with substance use disorders were identified using the same data source as for mental disorders, as well as data on treatment from the Registry of Drug Abusers Undergoing Treatment for the period 1996–2018 and supplemented with information from the National Registry of Alcohol Treatment during 2006–2018. In addition, we used information on drugs used to treat addictive disorders from the National Prescription Registry since 2015. Homeless people were identified using a previously developed algorithm, which primarily was based on the Homeless Register, with nationwide information on homeless shelter contacts in Denmark during 1999–2020.^13^ Persons who at some point during 1991–2020 had been imprisoned were identified from the Central Criminal Register.

### Ascertainment of infection with SARS-CoV-2

Individuals with a SARS-CoV-2 infection were identified as having had a PCR-confirmed SARS-CoV-2 infection. PCR tests were conducted as throat swabs in any of the free-of-charge test stations in Denmark and results were retrieved from the national Danish Microbiology Database, with the last update on 15 October 2021.^19,21^ Individuals were considered as infected from the date of a first record of being SARS-CoV-2 positive until the end of the follow-up. In addition, we considered people who had been admitted to a general hospital for more than 12 h and within 14 days of the date of a positive PCR test as having a SARS-CoV-2 infection-related hospital admission.^14^

### Outcome

Owing to the fact that self-harm events are under-recorded in Danish registries,^29^ we opted to examine a broader algorithm of hospital contacts that might be considered probable self-harm. This category covers self-harm regardless of intent, and includes poisoning by drugs and biological and non-medical substances, as well as lesions to the hand and forearm. Self-harm episodes were identified as presentations to either psychiatric or general hospitals, including emergency departments, and recorded in the National Patient Registry with one of the following ICD diagnoses: ICD-10 X60–X84, or where the reason for contact was coded as being suicide attempt (ALCC04) or non-suicidal self-harm (ALCC05). In addition, the following combinations of ICD diagnoses were included: a main diagnosis of a mental disorder (ICD-10 F00–F99) together with one of the following subdiagnoses: S51, S55, S59, S61, S65, S69 (cutting by sharp objects), T36–T50 (poisoning by pharmaceuticals), T52–T60 (poisoning by non-pharmaceuticals) as well as all admissions with a main diagnosis of T39, T40 (poisoning by mild analgesics; except T40.1), T42, T43 and T58 (poisoning by opioids, psychotropics or carbon monoxide). Individuals who died on the same or subsequent day as the record of the self-harm were considered as having died by suicide and excluded. The same measure has been used previously.^29^

As a sensitivity analysis, we restricted the outcome to consist only of self-harm episodes that were exclusively identified as diagnoses (ICD-10 X60–X84) or where the reason for contact was coded as being a suicide attempt (ALCC04).

### Follow-up

Participants were followed from 27 February 2020 to date of first subsequent self-harm episode, migration out of the country, death or 15 October 2021, whichever occurred first.

### Statistical analyses

Poisson regression analysis was used to calculate adjusted incidence rate ratios (aIRRs) with 95% confidence intervals (CIs) per 100 000 person-years, where rates of self-harm for individuals with a positive PCR test for SARS-CoV-2 infection were calculated relative to individuals not recorded with a positive test. The outcome was defined as a first incident of a self-harm episode since onset of the pandemic. When assessing rates in relation to SARS-CoV-2 infection-related hospital admission, models were further stratified by history of mental disorder and other hospital admissions (general and psychiatric combined). In addition, self-harm rates of high-risk groups were analysed in models with high-risk status and PCR-confirmed SARS-CoV-2 infection. Analyses were adjusted for calendar time (months), age (5-year groups), gender (female, male) and country of origin (Denmark, other high-income country or low- and middle-income country). Homelessness and imprisonment were handled as time-dependent variables, whereas all other covariates were fixed as of 27 February 2020. The association between SARS-CoV-2 and self-harm was tested in a sensitivity analysis where the outcome was restricted to a more conservative definition of self-harm, as described above.

Cumulative incidences of self-harm were estimated for individuals followed from the date of a first PCR-confirmed SARS-CoV-2 infection and for the subsequent 9 months in a subsample where each case was matched on age and gender to two individuals from the general population who had not been recorded with a positive test on the date of matching. Using the Aalen–Johansen estimator, we calculated the cumulative incidence of self-harm, while accounting for competing risks from death and emigration.

The statistical analyses were performed using SAS software for Windows (version 9.4).

### Ethical considerations

The project was approved by the Danish Data Protection Agency (Capital Region of Denmark: P-2020-439), Statistics Denmark and the Danish Health Data Authority. Data access was agreed by Statistics Denmark and the Danish Health Data Authority. Approval by an ethics committee and written informed consent were not required for this register-based project, according to Danish regulations. All data were de-identified and not recognisable at an individual level.

## Results

A total of 4 412 248 individuals (50.6% females) were observed over 7 069 961 person-years (mean follow-up: 1.6 person-year). Between 27 February 2020 and 31 October 2021, 260 663 (5.9%) individuals tested positive for SARS-CoV-2 infection with a PCR test, at a median age of 40.3 years (5th percentile: 16.7 years; 95th percentile: 75.5 years).

Of 5453 first records of a self-harm episode, 131 (2.4%) were for individuals who had previously tested positive for SARS-CoV-2 infection, resulting in an incidence rate of 74.7 (95% CI 61.9–87.5) per 100 000 person-years compared with a rate of 77.2 (95% CI 75.1–79.3) per 100 000 person-years in the remaining population (Supplementary Table 3). When adjusted for calendar time, age, gender and country of origin, individuals with SARS-CoV-2 infection had an aIRR for self-harm of 0.86 (95% CI 0.72–1.03; *P* = 0.09) compared with the remaining population.

### SARS-CoV-2 infection-related hospital admission

We identified 12 834 individuals who had a SARS-CoV-2 infection*-*related hospital admission to a general hospital (median age 68.2 years; 5th percentile: 30.9 years; 95th percentile: 90.0 years). Individuals with a SARS-CoV-2 infection*-*related hospital admission had a self-harm rate of 178.2 per 100 000 person-years, whereas those with a SARS-CoV-2 infection that did not result in hospital admission had a rate of 68.0 (Table 1). The self-harm rate among individuals with no SARS-CoV-2-positive PCR test and no record of being admitted to hospital during follow-up was 55.1 per 100 000 person-years, whereas those admitted to a general hospital for other reasons had a rate of 363.0. Compared with people not infected and not admitted to hospital, individuals with a SARS-CoV-2 infection*-*related hospital admission had an aIRR of 6.45 (95% CI 4.10–10.15), which was comparable to those with no positive test and a hospital admission for other reasons, who had an aIRR of 10.27 (95% CI 9.65–10.93).

**Table 1:** Incidence rates and adjusted incidence rate ratios of self-harm by population group in combination with SARS-CoV-2 infection and hospital admission

	*n*/*N*	Incidence rate per 100 000 person-years	aIRR (95% CI)^a^
Total	5453/4 412 248	77.1	
General population			
No SARS-CoV-2 infection, no hospital admission	3529/3 602 350	55.1	1 (ref.)
No SARS-CoV-2 infection, hospital admission	1793/549 235	363.0	10.27 (9.65–10.93)
SARS-CoV-2 infection, no hospital admission	72/224 624	47.2	0.84 (0.67–1.07)
SARS-CoV-2 infection, hospital admission	40/23 205	332.4	7.68 (5.61–10.51)
SARS-CoV-2 infection-related hospital admission	19/12 834	178.2	6.45 (4.10–10.15)
No history of mental disorder			
No SARS-CoV-2 infection, no hospital admission	1639/3 121 328	29.7	1 (ref.)
No SARS-CoV-2 infection, hospital admission	446/430 096	116.6	4.81 (4.29–5.39)
SARS-CoV-2 infection, no hospital admission	48/198 295	35.7	0.90 (0.67–1.21)
SARS-CoV-2 infection, hospital admission	16/18 435	169.1	5.34 (3.25–8.76)
SARS-CoV-2 infection-related hospital admission	5/9990	60.0	2.52 (1.05–6.09)
History of mental disorder			
No SARS-CoV-2 infection, no hospital admission	1890/481 022	213.3	1 (ref.)
No SARS-CoV-2 infection, hospital admission	1347/119 139	1207.5	9.16 (8.50–9.88)
SARS-CoV-2 infection, no hospital admission	24/26 329	131.8	0.81 (0.54–1.22)
SARS-CoV-2 infection, hospital admission	24/4770	933.9	7.15 (4.76–10.72)
SARS-CoV-2 infection-related hospital admission	14/2844	600.3	7.14 (4.21–12.11)
SARS-CoV-2, hospital admission, history of mental disorder^b^			
No SARS-CoV-2 infection, no hospital admission, no mental disorder	1639/3 121 328	29.7	1 (ref.)
No SARS-CoV-2 infection, no hospital admission, mental disorder	1890/481 022	213.3	6.91 (6.46–7.38)
No SARS-CoV-2 infection, hospital admission, no mental disorder	446/430 096	116.6	6.06 (5.44–6.76)
No SARS-CoV-2 infection, hospital admission, mental disorder	1347/119 139	1207.5	55.95 (51.88–60.34)
SARS-CoV-2 infection, no hospital admission, no mental disorder	48/198 295	35.7	1.15 (0.86–1.53)
SARS-CoV-2 infection, no hospital admission, mental disorder	24/26 329	131.8	4.59 (3.06–6.88)
SARS-CoV-2 infection, hospital admission, no mental disorder	16/18 435	169.1	7.04 (4.30–11.54)
SARS-CoV-2 infection, hospital admission, mental disorder	24/4770	934.0	39.65 (26.45–59.44)
SARS-CoV-2 infection-related hospital admission, no mental disorder	5/9999	600.0	3.72 (1.54–8.95)
SARS-CoV-2 infection-related hospital admission, mental disorder	14/2844	600.3	36.67 (21.63–62.16)

aIRR, incidence rate ratio; hospital admission, any hospital admission during follow-up; SARS-CoV-2 infection-related hospital admission, an admission with a medical disorder with duration of more than 12 h within 14 days of a PCR-confirmed SARS-CoV-2 infection; ref., reference value.

a. Model is adjusted for: calendar time (months), age (5-year age groups), gender and country of origin.

b. The *P*-value for the interaction was 0.55.

When stratifying this group by history of mental disorder, we found that rates of self-harm among SARS-CoV-2-positive individuals with no history of mental disorder were comparable, irrespective of whether hospital admission had been SARS-CoV-2 related or not (SARS-CoV-2 hospital admission: aIRR = 2.52; 95% CI 1.05–6.09; other hospital admission: aIRR = 5.34; 95% CI 3.25–8.76) and when compared with those with no SARS-CoV-2 infection and no hospital admission. Among individuals with a history of mental disorder and a positive test, rates of self-harm were also comparable with respect to SARS-CoV-2-related or other hospital admissions (SARS-CoV-2 hospital admission: aIRR = 7.14; 95% CI 4.21–12.11; other hospital admission: aIRR = 7.15; 95% CI 4.76–10.72). Individuals with SARS-CoV-2 but no hospital admission had comparable rates of self-harm to individuals with no SARS-CoV-2. In a model where presence of mental disorder and hospital admission were examined jointly, a higher rate of self-harm was noted among individuals with mental disorders and SARS-CoV-2-related hospital admission (aIRR = 36.67; 95% CI 21.63–62.16) but also among individuals with mental disorders and hospital admission not related to SARS-CoV-2 (aIRR = 39.65; 95% CI 26.45–59.44) when compared with those with neither.

### High-risk groups

No significant difference in self-harm rates was found for persons who had completed only primary school or less with respect to SARS-CoV-2 infection (*n* = 1 272 657; SARS-CoV-2 infection aIRR = 2.66, 95% CI 2.11–3.36 versus no SARS-CoV-2 infection aIRR = 2.91, 95% CI 2.74–3.10; *P* = 0.67) when compared with people with higher educational attainment and no positive test (Table 2). Comparable aIRRs were also found for individuals with chronic medical conditions (*n* = 2 213 834; SARS-CoV-2 infection aIRR = 1.58, 95% CI 1.22–2.05 versus no SARS-CoV-2 infection aIRR = 2.15, 95% CI 2.03–2.29; *P* = 0.084) and disability pension (*n* = 188 654; SARS-CoV-2 infection aIRR = 3.59, 95% CI 1.79–7.20 versus no SARS-CoV-2 infection aIRR = 6.57, 95% CI 6.06–7.13; *P* = 0.10) when compared with non-exposed individuals.

**Table 2:** Incidence rates and incidence rate ratios of self-harm by population group in combination with PCR-confirmed SARS-CoV-2 infection status

	*n*/*N*	Incidence rate per 100 000 person-years	aIRR^a^ (95% CI)	*P*^b^
Total	5453/4 412 248	77.1		
SARS-CoV-2 infection				
None	5322/4 151 585	77.2	1 (ref.)	0.09
Yes	131/260 663	74.7	0.86 (0.72–1.03)	
Low educational level				
None (higher educational level)	2305/2 886 129	47.6	1 (ref.)	0.83
SARS-CoV-2 infection	54/172 664	43.4	0.85 (0.65–1.11)	
Lower educational level	3017/1 265 456	147.0	3.04 (2.86–3.24)	
Both	79/87 999	142.1	2.76 (2.19–3.47)	
Chronic medical condition				
None	2158/2 046 135	63.7	1 (ref.)	0.08
SARS-CoV-2 infection	71/152 279	72.0	1.00 (0.79–1.26)	
Chronic medical condition	3164/2 105 450	90.2	2.15 (2.03–2.29)	
Both	60/108 384	78.2	1.58 (1.22–2.05)	
Disability pension				
None	4455/3 970 796	67.6	1 (ref.)	0.10
SARS-CoV-2 infection	123/252 798	72.4	0.95 (0.79–1.14)	
Disability pension	867/180 789	287.9	6.57 (6.06–7.13)	
Both	8/7865	146.7	3.59 (1.79–7.20)	
Mental disorder				
None	2085/3 551 424	35.4	1 (ref.)	0.06
SARS-CoV-2 infection	69/226 720	45.3	1.12 (0.88–1.43)	
Mental disorder	3,237/600 161	324.4	8.93 (8.45–9.44)	
Both	62/33 943	268.4	7.16 (5.55–9.23)	
Severe mental illness				
None	3452/3 927 009	52.9	1 (ref.)	0.26
SARS-CoV-2 infection	101/249 601	60.2	0.97 (0.80–1.19)	
Severe mental illness	1870/224 576	499.9	10.98 (10.36–11.64)	
Both	30/11 062	390.7	8.47 (5.90–12.14)	
Substance use disorder				
None	3421/3 852 406	53.5	1 (ref.)	0.01
SARS-CoV-2 infection	112/248 510	67.0	1.05 (0.87–1.27)	
Substance use disorder	1901/299 179	382.4	9.86 (9.30–10.47)	
Both	19/12 153	233.1	5.55 (3.53–8.72)	
Homelessness				
None	4891/4 117 969	71.5	1 (ref.)	0.33
SARS-CoV-2 infection	126/259 053	72.3	0.91 (0.76–1.08)	
Homelessness	431/33 616	774.9	14.54 (13.14–16.10)	
Both	5/1610	472.1	8.68 (3.60–20.89)	
Imprisonment				
None	4884/4 067 935	72.3	1 (ref.)	0.58
SARS-CoV-2 infection	125/256 402	72.4	0.89 (0.74–1.07)	
Imprisonment	438/83 650	313.9	7.09 (6.39–7.87)	
Both	6/4261	226.2	5.03 (2.26–11.23)	

aIRR, incidence rate ratio; ref., reference value; chronic medical condition, any chronic medical condition diagnosed prior to 27 February 2020; severe mental disorder, any severe mental illness (schizophrenia, bipolar disorder or depressive disorder) diagnosed prior to 27 February 2020; substance use disorder, any alcohol or drug use disorder diagnosed prior to 27 February 2020; homelessness, any history of homeless shelter contact; imprisonment, any history of imprisonment.

a. Model is adjusted for: calendar time (months), age (5-year age groups), gender and country of origin.

b. *P*-value to test for interaction between SARS-CoV-2 infection and examined subgroup, except for the first model, which examined rates of self-harm in relation to SARS-CoV-2 infection only.

Self-harm rates of individuals with a history of mental disorder did not vary with respect to whether these individuals had a confirmed SARS-CoV-2 infection or not (*n* = 634 104; SARS-CoV-2 infection aIRR = 7.16, 95% CI 5.55–9.23 versus no SARS-CoV-2 infection aIRR = 8.93, 95% CI 8.45–9.44; *P* = 0.06) when compared with those with neither. Similar rates of self-harm were also observed among individuals with severe mental illness whether or not they had a confirmed SARS-CoV-2 infection. Individuals with a substance use disorder in combination with SARS-CoV-2 infection had lower rates of self-harm than those with a substance use disorder and no positive SARS-CoV-2 test (*n* = 311 332; SARS-CoV-2 infection aIRR = 5.55, 95% CI 3.53–8.72 versus no SARS-CoV-2 infection aIRR = 9.86, 95% CI 9.30–10.47; *P* = 0.008). Among individuals with a history of homelessness or imprisonment, those with a positive SARS-CoV-2 test and those with no such record were found to have comparable self-harm rates.

### Cumulative incidence rates

**Fig. 1:**
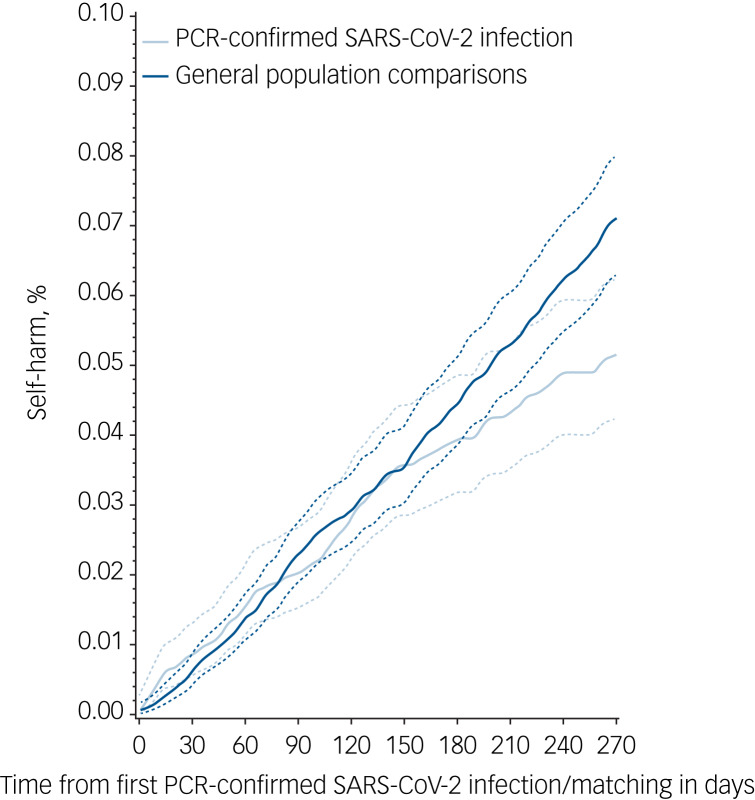
Cumulative incidence with their 95% confidence intervals of self-harm in people with PCR-confirmed SARS-CoV-2 infection compared with matched controls from the general population without a PCR-confirmed SARS-CoV-2 infection at the time of matching.

**Fig. 2:**
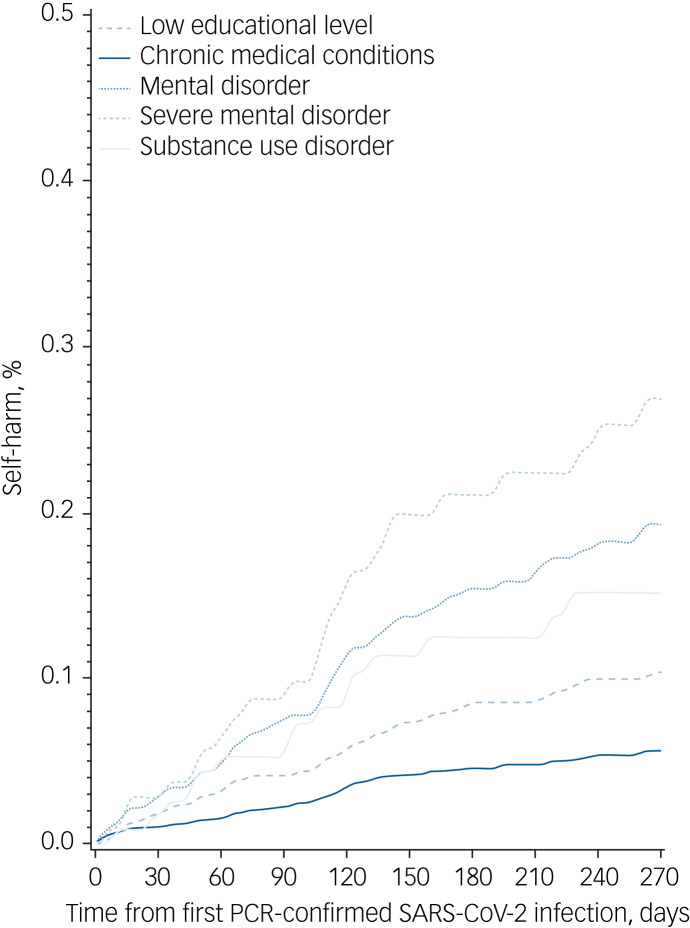
Cumulative incidences of self-harm status for subgroups. Curves are not shown for disability pension, homelessness and imprisonment as there were too few cases.

After 9 months of follow-up, the cumulative incidence rate for self-harm was 0.05% (95% CI 0.04–0.06) for those who had tested positive on a PCR test for SARS-CoV-2 infection and 0.07% (95% CI 0.06–0.08) for gender- and age-matched controls without a PCR-confirmed SARS-CoV-2 infection (Fig. 1). At the end of follow-up, the corresponding figures were 0.08% (95% CI 0.06–0.12) for those with SARS-CoV-2 infection and 0.12% (95% CI 0.10–0.15) for those without (Supplementary Table 4). The highest cumulative incidences of self-harm were found among individuals who had tested positive for SARS-CoV-2 infection and had severe mental illness (cumulative incidence 0.50%; 95% CI 0.29–0.81), a history of homelessness (cumulative incidence 0.48%; 95% CI 0.18–1.12) and/or substance use disorder (cumulative incidence 0.42%; 95% CI 0.15–0.99). As seen in Fig. 2, individuals with chronic medical conditions had the lowest cumulative incidence. The probability of self-harm during the study period for the entire population and by gender are shown in Supplementary Fig. 1.

Overall, when restricting the outcome to individuals recorded with a definite self-harm episode (i.e. excluding those with only a suggestive marker for self-harm), a positive PCR test for SARS-CoV-2 infection was associated with reduced risk of self-harm (aIRR = 0.63, 95% CI 0.43–0.91) compared with those with no positive test, after adjusting for gender, age, calendar time and country of origin.

## Discussion

Having nationwide and individual-level data on all tests conducted in Denmark allowed us to identify all individuals with a PCR-confirmed SARS-CoV-2 infection. SARS-CoV-2 infection was not found to be associated with self-harm in adjusted analyses. In fact, a lower rate of self-harm was found among those with SARS-CoV-2 infection when further restricting the definition of self-harm. Individuals admitted to hospital for SARS-CoV-2 infection-related disorders had an elevated rate of self-harm compared with those with no confirmed SARS-CoV-2 infection or hospital admission. However, their rate was comparable to that for individuals with SARS-CoV-2 who were admitted to hospital for other reasons. Among individuals with a history of mental disorder, we found elevated self-harm rates for those with a SARS-CoV-2 infection-related hospital admission as well as for those admitted to hospital with other physical or mental conditions. Although higher rates of self-harm were found among people who had had a SARS-CoV-2 infection and lower educational attainment, chronic medical conditions, history of mental disorder or history of homelessness or imprisonment compared with the remaining population, these rates were comparable to those for peers who had not had a confirmed SARS-CoV-2 infection. For substance use disorder, the rate of self-harm was higher in those without than those with a PCR-confirmed SARS-CoV-2 infection.

A high level of trust in the authorities, free-of-charge tests and easily accessible test sites are believed to have contributed to the high SARS-CoV-2 testing rate in Denmark.^13,30^ There was, however, also a high compliance with SARS-CoV-2 vaccination and 87% of the population received two doses of the vaccine during this follow-up period,^13^ which might have lessened the burden of the disease.

Contrary to our expectation, we found that a history of PCR-confirmed SARS-CoV-2 infection was associated with reduced risks of self-harm. When assessing self-harm using the more restrictive definition, we found a lower rate of self-harm. Individuals with a positive PCR test may only have been affected by the SARS-CoV-2 infection for a limited time. Owing to the short study period, we opted to consider individuals exposed for a longer period although this might have underestimated a possible effect. Analogous to clinical reports of excess prevalence of depressive symptoms and anxiety among individuals in hospital treatment for SARS-CoV-2,^31^ we found a substantially higher rate of self-harm among individuals with a SARS-CoV-2 infection*-*related hospital admission than for those not admitted to hospital. However, a higher rate of self-harm was also found among those with a positive PCR test and a later hospital admission that was assumed not to be related to SARS-CoV-2. Mental and physical disorders have previously been linked to self-harm,^32^ and it is possible that the increased risk relates to the level of distress experienced by individuals with any disorder that necessitates hospital admission, rather than SARS-CoV-2 itself. Still, individuals with mental disorders who were admitted to hospital because of SARS-CoV-2 were found to have higher rates of self-harm than individuals with no mental disorders who were admitted because of SARS-CoV-2, suggesting that there may have been an extra vulnerability for those with mental disorders. Although it was beyond the scope of the present study, it is possible that people suffering from long-term consequences of a SARS-CoV-2 infection may experience an excess risk of self-harm.

High rates of suicidal behaviour have previously been shown for vulnerable groups, such as individuals with chronic medical conditions, severe mental illness and homelessness.^9–12^ An excess risk of self-harm was not found for these individuals when they had a confirmed SARS-CoV-2 infection. It is possible that specific subgroups, such as those with chronic medical conditions or SARS-CoV-2 infection in general, received an increased level of informal support from their social network during the pandemic and when infected. On the other hand, access to ongoing treatment and other forms of formal support was probably interrupted or compromised during periods when individuals had a SARS-CoV-2 infection.^33^ Previous findings from Denmark showed that individuals with mental disorders, substance use disorder or experiences of homelessness or imprisonment were less likely to be PCR tested than those without these characteristics,^14^ which might introduce selection bias.

We cannot exclude the possibility that the lower rate found among individuals with SARS-CoV-2 infection in the sensitivity analysis might be due to an underdiagnosis of self-harm in select subgroups, for instance individuals with substance use disorder. Nevertheless, the result supports the interpretation that SARS-CoV-2 infection was not associated with a higher risk of self-harm.

### Strengths and limitations

Strengths of our study include the use of national administrative registers, thus minimising the risk of potential selection bias. These included complete and individual-level PCR test results, which were free of charge, resulting in high testing rates.^34^ Having longitudinal data, only self-harm episodes that took place after the date of a positive PCR test were considered as having occurred after exposure to a SARS-CoV-2 infection. In addition, we were able to obtain data for high-risk groups, such as socially marginalised people with a history of homelessness and imprisonment. Having complete data on all hospital admissions allowed us to identify individuals with severe SARS-CoV-2 infection. Applying a cohort design to data for the entire country enabled us to generate nationally representative findings. By adjusting for relevant confounders, we minimised bias.

Limitations of our study include the fact that it is possible that not all individuals who experienced symptoms were tested or some might have used rapid tests instead of going to a testing station, which is likely to have made our estimates conservative. Yet, it was only at the end of 2021 that self-testing kits became widely available in Denmark. Given that the follow-up ended before the massive wave of SARS-CoV-2 infections caused by the transition to the Omicron variant of concern,^35^ our findings are not representative for that period. Individuals with substance use disorder or homeless persons might have been less inclined to go for testing, which might imply some bias in those estimates. Although the wider definition of self-harm, which has previously been used in other investigations,^29^ is likely to capture more episodes, some may have been accidents. Also, the pandemic might have deterred individuals from seeking hospital care after self-harm, resulting in an under-recording of self-harm episodes. It is possible that members of the examined study subgroups were more (or less) inclined to seek hospital care for self-harm than others, which could bias our estimates, e.g. if they died. It is also possible that they received more social support during the pandemic. We did not have confirmative information that a subsequent general hospital admission was due to SARS-CoV-2. The small numbers prevented us from examining people who were currently experiencing homelessness or imprisonment. Potentially relevant but not included factors, such as level of informal support or previous self-harm, might have acted as confounders.

This national study with complete data on all individuals PCR-tested for SARS-CoV-2 infections did not find higher self-harm rates among individuals with PCR-confirmed SARS-CoV-2 infection; rather, rates were found to be at the level of those with no positive tests. We did find higher rates of self-harm among high-risk individuals with a hospitalisation but this was irrespective of whether there wa a relation to a SARS-CoV-2 infection or not.

## Data Availability

The data that support the findings of this study are available from Statistics Denmark. Data access requires the completion of a detailed application form from the Danish Data Protection Agency, the Danish National Board of Health and Statistics Denmark. For more information on accessing the data, see https://www.dst.dk/en.

## Supplementary material

For supplementary material accompanying this paper visit https://doi.org/10.1192/bjp.2022.194. click here to view supplementary material
