# Supplementary material for: Association between SARS-CoV-2 infection and self-harm: Danish nationwide register-based cohort study
Source: Br J Psychiatry. 2023 Apr;222(4):167–74. doi: 10.1192/bjp.2022.194 (PMC10895503; doi:10.1192/bjp.2022.194)
Supplement: Supplementary file 1 [file S0007125022001945sup001.docx]

Supplementary Figure 1. Cumulative incidence of self-harm in the Danish general population overall and by sex, Feb 27, 2020 to Oct 15, 2021

1. **Overall^*^**

**
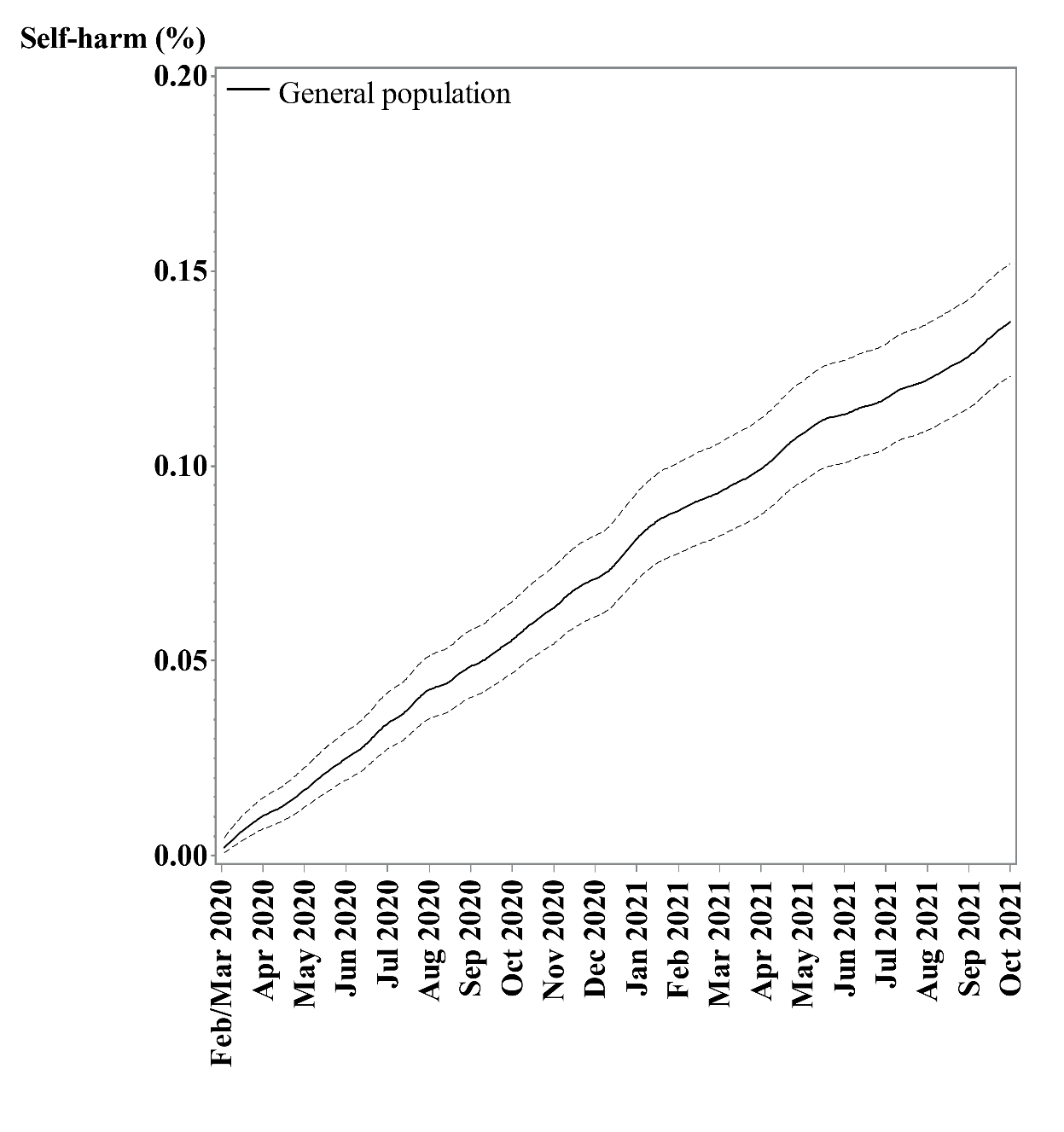
**

^*^At end of follow-up, i.e., at Oct 15, 2021, the cumulative incidence was 0.12 (95% CI 0.11-0.12).

**B. Sex**^‡^

**
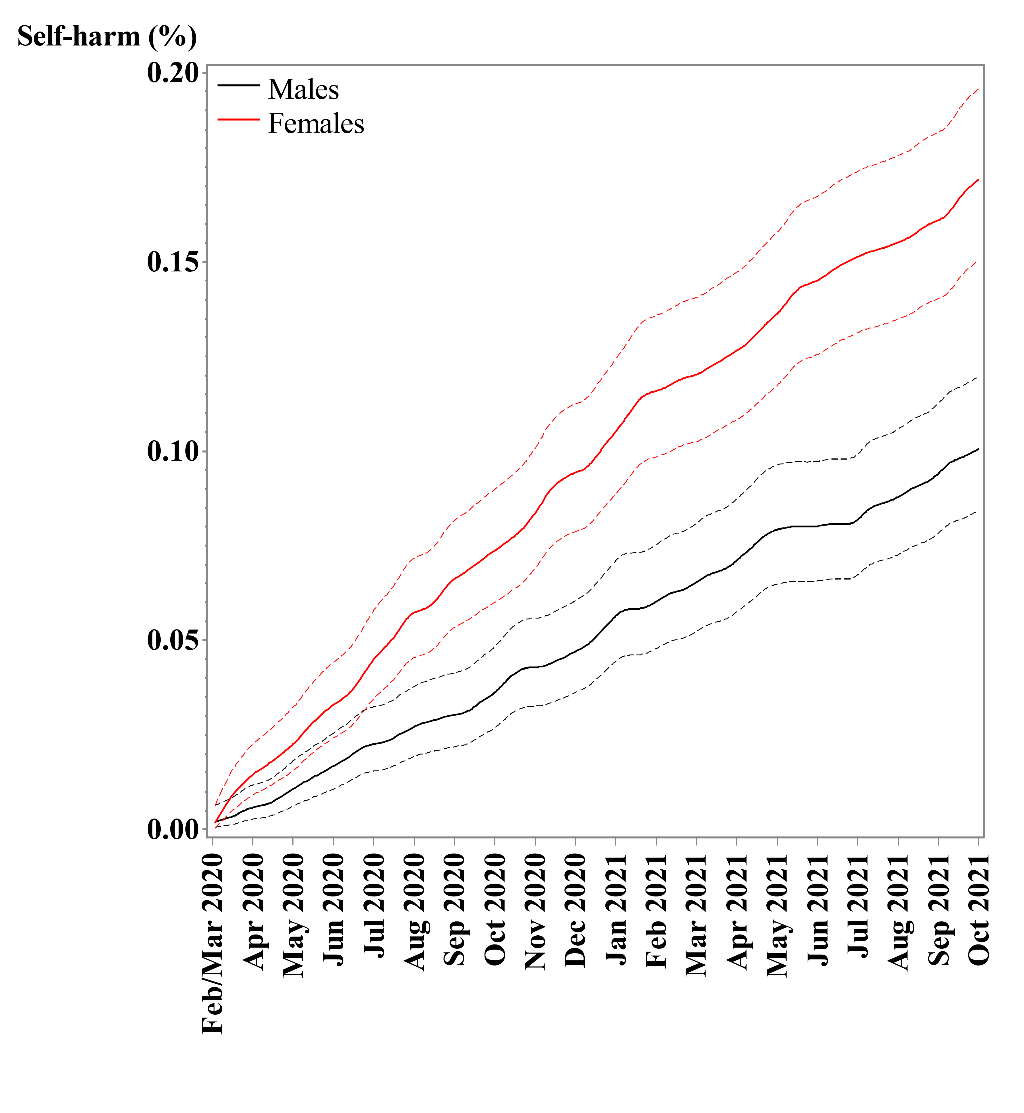
**

^‡^At end of follow-up, i.e., at Oct 15, 2021, the cumulative incidence was 0.09 (95% CI 0.09-0.10) and 0.14 (95% CI 0.14-0.15) for males and females, respectively.

Supplementary Table 1. Definitions of high-risk groups.

| **Measure** | **Categories** | **Period** | **Data sources** | **Classification system and codes** | **Data authority** |
| --- | --- | --- | --- | --- | --- |
| Educational level | - Primary school degree - Higher than Primary school (missing values included) | 2019 | Register data on highest obtained educational level from the Population Education Register | International Standard Classification of Education | Statistics Denmark |
| Disabilty pension | - Working - Unemployed - Disability pension - Pension - Other/missing | 2019 | The Employment Classification Module (Petersson et al., 2011) | Statistics Denmark constructs a variable on each indivdual's socio-economic status based on national register data on income sources and employment. | Statistics Denmark |
| Mental disorder | - Any mental diagnosis prior to study start  - No mental diagnosis prior to study start | 1969-2020 (Dec 27) | The Danish Psychiatric Central Research(Mors et al., 2011b) Register/The Danish National Patient Register(Schmidt et al., 2015) | ICD-8: 290-315 (excl. 291.x9, 303.x9, 303.20, 303.28, 303.90 571.00, 571.01, 294.39, 304.x9) ICD-10: F00, F20-F99 | The Danish Health Data Authority |
| Severe mental disorder | - Any severe mental diagnosis prior to study start  - No severe mental diagnosis prior to study start | 1969-2020 (Dec 27) | The Danish Psychiatric Central Research(Mors et al., 2011b) Register/The Danish National Patient Register(Schmidt et al., 2015) | ICD-8: 295.09, 295.19, 295.29, 295.39, 295.49, 295.59, 295.69, 295.89, 295.99, 296.19, 296.39, 298.19, 296.09, 296.29, 298.09, 300.49 ICD-10: F20, F30-31, F32-33 | The Danish Health Data Authority |
| Substance misuse | - Any substance use disorder prior to study start   - No substance use disorder prior to study start | 1969-2020 (Dec 27) | The Danish Psychiatric Central Research Register/The Danish National Patient Register/The Danish National Prescription Registry(Pottegård et al., 2017)/The National Registry of Alcohol Treatment/The Registry of Drug Abusers Undergoing Treatment | **ICD-8:** 291.x9, 303.x9, 303.20, 303.28, 303.90, 571.00, 571.01, 294.39, 304.x9  **ICD-10:** F10-F19 **ATC:** N07BB (alcohol dependence), N07BC (opiods dependence) | The Danish Health Data Authority |
| Homelessness | - Any experience of homelessness  - No experience of homelessness | 1996-2020 | Postal address^*^/The Danish Homeless Register(Statistics Denmark, 2020)/The Registry of Drug Abusers Undergoing Treatment (National Board of Health, 2020b)/The National Registry of Alcohol Treatment(National Board of Health, 2020a) | The Act of Social Services §110 and self-reported homelessness | Statistics Denmark/ The Danish Health Data Authority |
| History of imprisonment | - Any history of imprisonment  - No history of imprisonment | 1991-2020 | The Central Criminal Register | Any record of imprisonment during 1991-2000 | Danish National Police |

*Having a postal address during 2018-2020 was defined as the period from Jan 1, 2018 and until Nov 11, 2020 (latest update). The addresses from the “Tilbudsportalen” were validated by corresponding street codes in Denmark.

Supplementary Table 2. Algorithm for chronic medical health conditions.

| **Category** | **Disease group** | **Definition** | **ICD-10 Codes** | **Drug codes (ATC) (Time frame for prescriptions)** |
| --- | --- | --- | --- | --- |
| **Any chronic medical condition^*^** | |  |  |  |
| **Circulatory system** | |  |  |  |
|  | Hypertension | Diagnosis AND/OR prescriptions of antihypertensives**^†^** | I10-I13, I15 | C02, C03, C04, C07, C08, C09 (Twice in 1 year) |
|  | Dyslipidemia | Diagnosis AND/OR prescription of lipid-lowering drugs**^‡^** | E78 | C10 (Twice in 1 year) |
|  | Ischemic heart disease | Diagnosis AND/OR prescription for antianginal drug | I20-I25 | C01DA (Twice in 1 year) |
|  | Atrial fibrillation | Diagnosis | I48 |  |
|  | Heart failure | Diagnosis | I50 |  |
|  | Peripheral artery occlusive disease | Diagnosis | I70-I74 |  |
|  | Stroke | Diagnosis | I60-I64, I69 |  |
| **Endocrine system** | |  |  |  |
|  | Diabetes mellitus | Diagnosis AND/OR prescription of antidiabetics | E10-E14 | A10A, A10B (Twice in 1 year) |
|  | Thyroid disorder | Diagnosis AND/OR prescription of thyroid therapy drugs | E00-E05, E061-E069, E07 | H03 (Twice in 1 year) |
|  | Gout | Diagnosis | E79, M10 |  |
| **Pulmonary system and allergy** | | |  |  |
|  | Chronic pulmonary disease | Diagnosis AND/OR Prescription for obstructive airway disease drugs | J40-J47 | R03 (Twice in 1 year) |
|  | Allergy | Diagnosis AND/OR prescription for non-sedative antihistamines AND/OR nasal antiallergics | J30.1-J30.4, L23, L50.0, T78.0, T78.2, T78.4 | R06AX, R06AE07, R06AE09, R01AC, R01AD (Twice in 1 year) |
| **Gastrointestinal system** | |  |  |  |
|  | Ulcer/chronic gastritis | Diagnosis | K221, K25-K28, K293-K295 |  |
|  | Chronic liver disease | Diagnosis | B16-B19, K70, K74, K766, I85 |  |
|  | Inflammatory bowel disease | Diagnosis | K50-K51 |  |
|  | Diverticular disease of intestine | Diagnosis | K57 |  |
| **Urogenital system** | |  |  |  |
|  | Chronic kidney disease | Diagnosis | N03, N11, N18-N19 (ever) |  |
|  | Prostate disorders | Diagnosis AND/OR prescription of prostate hyperplasia therapy drugs | N40 | C02CA, G04C (Twice in 1 year) |
| **Musculoskeletal system** | | |  |  |
|  | Connective tissue disorders |  | M05-M06, M08-M09, M30-M36, D86 (ever) |  |
|  | Osteoporosis | Diagnosis AND/OR prescriptions for osteoporosis drugs | M80-M82 (ever) | M05B, G03XC01, H05AA (Twice in 1 year) |
|  | Painful condition | Repeated prescriptions of analgesics |  | N02A, N02BA51, N02BE, M01A, M02A (Four times in 1 year) |
| **Hematological system** | |  |  |  |
|  | HIV/AIDS | Diagnosis | B20-B24 |  |
|  | Anemias | Diagnosis | D50-D53, D55-D59, D60-D61, D63-D64 |  |
| **Cancers** |  |  |  |  |
|  | Cancer | Diagnosis | C00-C43, C45-C97 |  |
| **Neurological system** | |  |  |  |
|  | Vision problem | Diagnosis | H40, H25, H54 (ever) |  |
|  | Hearing problem | Diagnosis | H90-H91, H931 (ever) |  |
|  | Migraine | Diagnosis AND/OR prescription of specific anti-migraine drugs | G43 | N02C (Twice in 1 year) |
|  | Epilepsy | Diagnosis AND prescription of anti-epileptics | G40-G41 | N03 (Twice in 1 year) |
|  | Parkinson’s disease | Diagnosis | G20-G22 |  |
|  | Multiple sclerosis | Diagnosis | G35 |  |
|  | Neuropathies | Diagnosis | G50-G64 |  |
| ^*^The chronic medical conditions were defined according to previous studies^27, 36^ and were based on ICD-10 diagnoses recorded between Jan 1, 1994 and Dec 27, 2020 (study start). **^†^**Ascertained only in absence of ischemic heart disease/heart failure, and by diuretics only if no kidney disease. **^‡^**These prescriptions were used if no previous ischemic heart disease had been recorded. | | | | |

Supplementary Table 3: Study characteristics and risk of self-harm by PCR-confirmed SARS-CoV-2 infection status.

|  | SARS-CoV-2 infection | | |  | No SARS-CoV-2 infection | | |
| --- | --- | --- | --- | --- | --- | --- | --- |
|  | n/N | PY | IR (95% CI)  per 100,000 PY |  | n/N | PY | IR (95% CI)  per 100,000 PY |
|  |  |  |  |  |  |  |  |
| Overall | 131/260,663 | 175,352 | 74.7 (61.9-87.5) |  | 5322/4,151,585 | 6,894,609 | 77.2 (75.1-79.3) |
|  |  |  |  |  |  |  |  |
| Age (years) |  |  |  |  |  |  |  |
| 15-24 | 52/67,286 | 35,988 | 144.5 (105.2-183.8) |  | 1,548/574,259 | 839,699 | 184.4 (175.2-193.5) |
| 25-34 | 22/45,957 | 29,771 | 73.9 (43.0-104.8) |  | 865/549,910 | 921,749 | 93.8 (87.6-100.1) |
| 35-44 | 17/42,817 | 27,727 | 61.3 (32.2-90.5) |  | 598/576,312 | 957,365 | 62.5 (57.5-67.5) |
| 45-54 | 12/45,837 | 32,846 | 36.5 (15.9-57.2) |  | 753/702,186 | 1,171,263 | 64.3 (59.7-68.9) |
| 55-64 | 8/31,128 | 25,498 | 31.4 (9.6-53.1) |  | 640/665,590 | 1,131,225 | 56.6 (52.2-61.0) |
| 65-74 | 8/15,192 | 12,090 | 66.2 (20.3-112.0) |  | 459/602,659 | 997,021 | 46.0 (41.8-50.2) |
| 74+ | 12/12,446 | 11,432 | 105.0 (45.6-164.4) |  | 459/480,669 | 876,288 | 52.4 (47.6-57.2) |
|  |  |  |  |  |  |  |  |
| Sex |  |  |  |  |  |  |  |
| Female | 86/132,482 | 90,105 | 95.4 (75.3-115.6) |  | 3,225/2,100,040 | 3,489,986 | 92.4 (89.2-95.6) |
| Male | 45/128,181 | 85,247 | 52.8 (37.4-68.2) |  | 2,097/2,051,545 | 3,404,623 | 61.6 (59.0-64.2) |
|  |  |  |  |  |  |  |  |
| Country of origin |  |  |  |  |  |  |  |
| Denmark | 81/190,193 | 130,527 | 62.1 (48.5-75.6) |  | 4,673/3,639,686 | 6,029,381 | 77.5 (75.3-79.7) |
| Other Western Countries | 6/12,712 | 8,129 | 73.8 (14.7-132.9) |  | 194/204,443 | 325,444 | 59.6 (51.2-68.0) |
| Non-Western countries | 44/57,758 | 36,695 | 119.9 (84.5-155.3) |  | 455/307,456 | 539,786 | 84.3 (76.5-92.0) |

Abbreviations: PY: Person-years, IR: Incidence Rate.

Supplementary Table 4. Cumulative incidence of self-harm according to status at end of follow-up at Oct 15, 2021.^*^

|  | n | N | Cumulative incidence  on day 57 (95% CI) |
| --- | --- | --- | --- |
|  |  |  |  |
| SARS-CoV-2 infection |  |  |  |
| None | 313 | 502,151 | 0.12 (0.10-0.15) |
| Yes | 121 | 250,894 | 0.08 (0.06-0.12) |
|  |  |  |  |
| SARS-CoV-2 infection and lower educational level | 67 | 74,129 | 0.13 (0.10-0.17) |
|  |  |  |  |
| SARS-CoV-2 infection and chronic medical condition**^†^** | 71 | 132,983 | 0.10 (0.06-0.15) |
|  |  |  |  |
| SARS-CoV-2 infection and disability pension | 8 | 7,826 | 0.12 (0.06-0.23) |
|  |  |  |  |
| SARS-CoV-2 infection and mental disorder | 60 | 33,005 | 0.35 (0.22-0.56) |
|  |  |  |  |
| SARS-CoV-2 infection and severe mental disorders^‡^ | 30 | 10,944 | 0.50 (0.29-0.81) |
|  |  |  |  |
| SARS-CoV-2 infection and substance misuse§ | 19 | 12,049 | 0.42 (0.15-0.99) |
|  |  |  |  |
| SARS-CoV-2 infection and homelessness^¶^ | 5 | 1,568 | 0.48 (0.18-1.12) |
|  |  |  |  |
| SARS-CoV-2 infection and imprisonment^¶^ | 6 | 44,148 | 0.17 (0.07-0.35) |

^*^ Only individuals who were aged 15 years and over at start of the follow-up, i.e. Feb 27, 2020, were included in this analysis. **^†^**Each case was matched to two comparisons who were of same sex and age and without SARS-CoV-2 infection. For 44 cases, no comparison could be identified. ^‡^Defined as any pre-existing chronic medical condition diagnosed prior to Feb 27, 2020. ^§^Severe mental disorders defined as any pre-existing severe mental illness (schizophrenia diagnosis, bipolar disorder, or depressive disorder) diagnosed prior to Feb 27, 2020. ^¶^Substance misuse defined as any pre-existing alcohol or drug misuse diagnosed prior to Feb 27, 2020. ^**^Defined as any history of homelessness/imprisonment.
